# The ABCs of MGR with DCJ

**Published:** 2008-04-10

**Authors:** Zaky Adam, David Sankoff

**Affiliations:** 1 School of Information Technology and Engineering, University of Ottawa, Ottawa, Canada, K1N 6N5; 2 Department of Mathematics and Statistics, University of Ottawa, 585 King Edward Avenue, Ottawa, ON, Canada, K1N 6N5

## Abstract

We study the small phylogeny problem in the space of multichromosomal genomes under the double cut and join metric. This is similar to the existing MGR (multiple genome rearrangements) approach but it allows, in addition to inversion and reciprocal translocation, operations of transposition and block interchange. Empirically, with chloroplast and mammalian data sets, it finds solutions as good as or better than MGR when the latter operations are prohibited. Permitting these operations allows quantitatively better solutions where part of the reconstructed ancestral genomes may be included in circular chromosomes. We discuss the biological likelihood of transpositions and block interchanges in the mammalian data.

## 1. Introduction

In this paper we discuss a version of the small phylogeny problem in the metric space of multichromosomal genomes under a rearrangement distance metric. The particular metric we use is the double-cut-and-join metric (DCJ) [10]. This is similar to the existing MGR approach [2] but it allows, in addition to inversion and reciprocal translocation, operations of transposition and block interchange.

Models of genome rearrangement processes have permitted different repertoires of operations. Certainly, realistic models must account for inversion. They also must allow reciprocal translocations, and processes of chromosome fusion and fission, all of which involve transferring an entire telomeric (i.e. suffix or prefix) region of at least one chromosome.

Other movements of chromosomal fragments, usually not involving telomeres, are widely attested, and grouped together under the label of transpositions. They are produced by a variety of processes, such as gene duplication followed by the loss of the original copy, or retrotransposition, or recombination errors.

Of the three true movement rearrangements, inversion, translocation and transposition, only the first two, separately or in combination, have proved very amenable to mathematical modeling, as exemplified by the Hannenhalli-Pevzner formula for the edit distance between two genomes, i.e. the minimum number of operations required to transform one genome into another, and the efficient algorithm for producing such a series of operations. No formula or efficient algorithm exists for transposition, either by itself or in combination with the other two operations. As for other structural genome modifications, such as duplication of genes or of chromosomal segments, or deletions and insertions, while they are also aspects of genomic plasticity and often consequences or causes of movement rearrangements, mathematical models of rearrangement are not easily extended to encompass them.

Recently, Yancopoulos et al. [10] introduced the DCJ operation as the basis for generating all the movement rearrangements. This allowed for the inclusion of transposition with inversion and translocation in a single model and resulted in a simpler formula for the edit distance and a simpler algorithm for recovering a corresponding series of operations. A double cut and join operation simply cuts the chromosome in two places and joins the four ends of the cut in a new way.

The DCJ model, however, allows for the generation of a new kind of movement operation, a generalized transposition called block interchange, which is not represented in the biological genome rearrangement literature, though it has long been studied in the mathematical literature on rearrangement. Both transposition and block interchange can be thought of as the excision of a fragment, its circularization, together counting as one DCJ operation, followed by a second set of cuts, where the circle is not necessarily cut in the same place it was originally created through a join, and then reincorporated at a new site in the chromosome. Transpositions and block interchanges thus count as two DCJ operations whereas inversions and translocations each count as one.

We postpone the question of the biological significance of these chromosomal circles to Section 8. Yancopoulos et al.’s original publication [10] pointed out that the running time of their algorithm could be reduced to linear if circles were not constrained to be reincorporated into linear chromosomes as soon as they were generated. Bergeron et al. [1] recently restated the DCJ model and produced a simplified (linear) algorithm ignoring the reincorporation constraint and, as in the mathematical justification of DCJ in [10], without any explicit mention of the particular operations of inversion, translocation, transposition, interchange, fusion and fission. It is thus the most general existing algorithm for movement rearrangements. As it has a form which lends itself well to constraints on the operations allowed, it can largely emulate other algorithms, e.g. the Hannenhalli-Pevzner algorithm (but without taking into account “hurdles” and “knots”)or the Yancopoulos-Attie-Friedberg algorithm (at the cost of losing its computational efficiency).

Solutions of the small phylogeny problem in rearrangement metric spaces are generally based on iterations of a rearrangement median problem, namely the inference of an ancestral genome based on its three neighbours in a binary phylogeny. All indications are that the median problem in any rearrangement metric space is likely to be NP-hard [3,9]. Thus in Section 2 we present the general algorithm for basing a rearrangement phylogeny on the median problem, while in Section 3 we present a heuristic for the median problem in DCJ space. Section 4 discusses ways of avoiding local minima of the small phylogeny problem. The rest of the paper is devoted to applications to chloroplast and mammalian data sets.

## 2. The Small Phylogeny Problem under Rearrangement Distance

Given the quintuple (*N,P,n,𝒢,d*), where

Ę is a phylogeny with *N* labeled terminal nodes,

𝒢 is a set containing *N* genomes, each made up of 2*n* markers partitioned among one or more circularly or linearly ordered chromosomes; each marker is an ordered pair of form (*x*, *y*), where the “vertices” *x* and *y* represent the beginning and end of the marker; each genome is associated with one of the terminals of Ę, and

*d* is a metric (satisfying non-negativity, reflexivity, symmetry and the triangle inequality) on the set of all possible genomes with *n* markers.

The small phylogeny problem is to construct a set of genomes ℋ to associate with the non-terminal nodes of Ę, such that the phylogenetic tree length

(1) $$L(ℋ)=\sum_{XY\in ℬ}d(X,Y)$$

is minimal, where ℬ is the set of branches in Ę.

In this paper, we consider the simplest structure for Ę, namely an unrooted, binary-branching tree. All nodes are of degree one (terminals) or three (non-terminals). The overall structure of our (heuristic) algorithm for minimizing *L* is as follows:

### Algorithm Small Phylogeny

**input** Ę, 𝒢

set *L* = ∞

**Initialize** ℋ

calculate *L*′ = *L*(ℋ)

**while** *L*′ < *L*

 set *L* = *L*′

 choose an ordering of the elements of ℋ

 **for** each *G* ε ℋ, with neighbours *A*,*B*,*C*

  *G* = **Median** (*A*, *B*, *C*)

 *L*′ = *L*(ℋ)

**end while**

**Escape** from local minimum

**Output**

The initialization step can be important in reducing the computing time in the **while** loop and in the **Escape** routine. An easy initialization, but one which does not favour rapid convergence, consists of choosing a different random genome for each genome in ℋ. A better choice is to set the genome equal to the genome of one of the nearest terminal nodes.

The **Median** algorithm is the subject of Section 3.

The choice of ordering of ℋ is not of major importance. The order can be fixed at the outset once and for all, or it may change before each pass of ℋ in the hope of avoiding a poor local minimum.

The **Escape** routine is the subject of Section 4.

## 3. The Median Algorithm

We use the following notation to represent the adjacencies in a genome [1]. If two vertices *a* and *b* from different markers are adjacent in a genome, we represent this by an edge {*a,b*} = {*b,a*}; for a vertex *c* at the end of a chromosome and hence adjacent to no other vertex, we construct the singleton {*c*}. Then any rearrangement operation can be represented by an operation on one or two terms in the representation, such as {*a,b*}, {*c,d*} → {*b,d*}, {*a,c*} or {*a,b*} → {*b*}, {*a*} or {*a,b*}, {*c*} → {*b*}, {*a,c*}. **Algorithm Median** successively transforms all three genomes into their median.

This algorithm is inspired by the MGR algorithm [2] in its strategy of seeking operations which move each genome toward the other two as much as possible at each step. The details of which operations are prioritized are slightly different, as are the final steps towards the median. The use of the DCJ paradigm makes the coding straightforward, as can be deduced from the accompanying pseudocode. A consequence of the DCJ approach is that the median can contain circular chromosomes, even if the three neighbouring genomes have only linear chromosomes, whereas previous methods exclude the presence of circles in the median.

## 4. Escape from Local Minima

Once the small phylogeny algorithm converges, we seek a better minimum as follows. Again we iterate over all ancestral nodes until convergence. At each node *V*, we examine the adjacencies defining *V*’s current genome. Those adjacencies and singletons that are in all three or in any two of the neighbours constitute the invariant part of *V*.

Consider the set *U* containing just those adjacencies or singletons of *V* that are in only one of the neighbours. Our approach to finding a better minimum is to pick any two vertices at random in *U*, to perform a DCJ operation on the two adjacencies or singletons containing these two vertices and to add the resulting adjacencies or singletons to *U*, replacing the current adjacencies and singletons in *V*. If the resulting genome has better or equal median distance than the current minimum, it replaces the current genome. This is repeated a large number of times, 5000 in our experiments. When there is no longer any change in the total tree length, the algorithm terminates.

By retaining alternative medians of equal median distance at each step, this approach effectively searches far from the original solution. MGR [2] also includes a (somewhat different) post-processing step for escaping from local minima.

## 5. The Campanulaceae cpDNA Dataset

**Figure 1:**
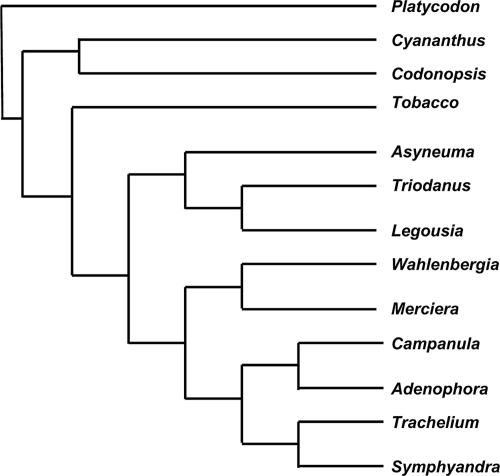
Phylogeny for Campanulaceae data set. Rooting and edge lengths arbitrary.

The well-known Campanulaceae chloroplast data-set consists of 13 cpDNAs with 105 markers each. Each genome consists of one circular chromosome. The data were first collected by E. Cosner and have been studied by Cosner et al. [4] and Moret et al. [7]. Using GRAPPA, Moret et al. reconstructed 216 tree topologies of Campanulaceae with a total distance of 67 reversals each. Bourque and Pevzner [2] used MGR to reconstruct one of these 216 trees, that shown in Figure 1, with a total distance of 65 inversions.

We ran our program on this data set using the tree reconstructed by MGR, without allowing the appearance of additional circular chromosomes (i.e. no transpositions or block interchanges), and obtained 64 DCJ operations. Running the program unconstrained, we obtained a total distance of 59 DCJ operations. Only four ancestors had an extra circular chromosome, but there is no biological evidence in the Campanulaceae, or other higher plants, of chloroplast genomes consisting of two or more circles.

### Algorithm Median

**input** three genomes with same gene content.

**while** it is possible to do an operation {*a,b*}, {*c,d*} → {*b,d*}, {*a,c*} that creates two adjacencies {*b,d*} and {*a,c*} in one genome that are already shared by the other two, execute such an operation.

**endwhile**

**while** it is possible to do an operation {*a,x*}, {*b,y*} → {*a,b*}, {*x,y*} that creates an adjacency {*a,b*} in one genome that is already shared by the other two, let *S* be the set of such operations. (N.B., either *x* or *y* or both may be null elements so that, e.g. {*a,x*} is just the singleton {*a*}.) For each operation in *S*, associate a score defined to be the increment in |S | were that operation to be applied. Choose an operation in *S* to apply with maximum positive score.

**if** |*S*| = 0 and it is possible to do operations {*a,x*}, {*b,y*} → {*a,b*}, {*x,y*} and {*a,w*}, {*b,z*} → {*a,b*}, {*w,z*} in two genomes that not only create an adjacency {*a,b*} in each that already exists in the third, but also create an element of *S*, execute such a pair of operations. (N.B., not all of *x,y,w* and *z* can be null.)

**else** if |*S*| = 0 and it is possible to do operations {*a,x*}, {*b,y*} → {*a,b*}, {*x,y*} and {*a,w*}, {*b,z*} → {*a,b*}, {*w,z*} in two genomes that create an adjacency {*a,b*} in each that already exists in the third, execute such a pair of operations that minimize the genomic distance between the two affected genomes. (N.B., not all of *x,y,w* and *z* can be null.)

**endif**

**endwhile**

**for** all adjacencies {*a,b*} in any genome where {*a*} and {*b*} are singletons in both other genomes, carry out the operation {*a,b*} → {*a*}, {*b*}.

**endfor**

(At this point all three genomes have been transformed to the same structure, the median.)

**for** all pairs of adjacencies and/or singletons in the median we carry out all possible DCJ operations on the pair and see if it reduces the sum of the distances between the median and the three original genomes; if so, we adjust the median accordingly. Whenever such an adjustment is found, we repeat this search.

**endfor**

**output** median genome.

## 6. Data Set on Mammals

**Figure 2:**
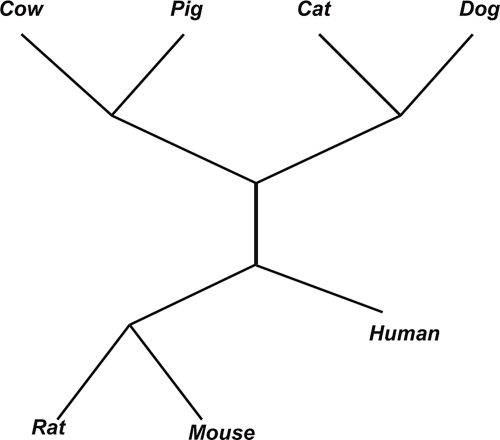
Phylogeny for mammalian data set. Rooting and edge lengths arbitrary.

The mammalian data set, drawn from [8], consists of the genomes of human, rat, mouse, cat, dog, pig and cow. Each genome consists of 307 HSB (homologous synteny blocks). In [8], the total distance of the tree in Figure 2 is 487 reversals, obtained using MGR.

Running DCJ on this data set using the same tree topology, without allowing the ancestors to have any circular chromosome also resulted in a total distance of 487 DCJ operations. The first local minimum was 495, but the **Escape** routine brought it down to 487. When we allowed ancestors to have circular chromosomes in addition to linear ones, we obtained a total distance of 486 DCJ operations. The number of circular chromosomes that appeared in each ancestor ranged between 1 and 5, but only in the immediate ancestors of the seven data species.

## 7. Implementation

Our experimental software was oriented to achieve the maximum accuracy through the **Escape** routine and the median improvement steps, with little regard to the size of problems beyond the ones considered here. However, there is much room for optimization of the code in view of larger data sets.

## 8. Evidence for Excision-Circularization-Linearization-Reincorporation

The DCJ approach can reconstruct circular chromosomes at speciation points although there is no current biological evidence for the durability over evolutionary time of circular chromosomes in the nuclear genomes of higher eukaryotes. While circularization is well-known and understood in the functioning of the immune system, in somatic cell tumors, classical “double minutes”, and various very small DNA molecules like episomes, and while ring chromosomes are a relatively common genetic abnormality, the existence of circular chromosomes as part of the normal genomic complement of a species, including in homozygotes and participating in normal meiosis, is unattested.

We have noted in our real examples, however, that when the DCJ operations are constrained, the algorithm produced solutions that are exactly as good as MGR solutions. This validates the suggestion in [1] that the notation and algorithm proposed in that article can serve as basis for exploring the effects of constraints on genome rearrangement problems.

The question remains, what is the evolutionary significance of these chromosomal circles, especially circular intermediates? Circular DNA structures abound in all sorts of organisms, even eukaryotes. Circular chromosomes are well-known in clinical studies [5] and the process of excision, circularization, linearization and reincorporation is exactly what happens in the configuration of the immune response in higher animals. And circular intermediates within germ line cells could play a role in rearrangement without becoming fixed in a population. But because the evolutionary consequences of block interchange could have come about in other ways, e.g. various combinations of nested inversions, there has been no reason to look for evidence of this process or even to notice it. The question of the existence or importance of block interchange remains open.

**Figure 3:**
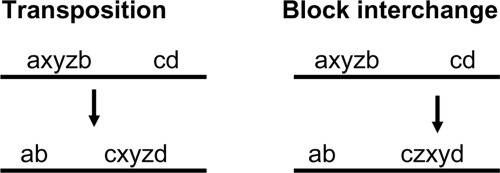
Diagnostics for transposition and block interchange. The “interchange” terminology can be understood from the interchange of *xyz* and *bc* in the transposition event and the interchange of *xy* and *bc* in the block interchange event.

How would we detect a transposition or a block interchange in closely related genomes? Figure 3 shows how the flanking markers of the transposed segment in one genome are adjacent in the other genome and vice versa. In genomes that are farther apart, we could expect some aspects of this pattern to be disrupted by subsequent rearrangements.

Still, a few of these may survive, or be clearly visible despite subsequent rearrangements. For example, one of the circular chromosomes at the ancestor of pig and cow in Figure 2 is made up of markers 127 and 128 in the numbering system of [8]. The segments at the beginning of chromosome 1 of pig, with the flanking segments outlined on either side of the boldface segments from the circular chromosome, are −137, −136, −135, −134, 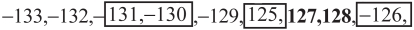 271, 272, …, while chromosome 9 of cow is 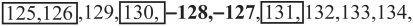 135, 136, 137. This fits the pattern for transposition in Figure 3, aside from the subsequent inversion of segment 126.

Segments 205 and 206 can form another circle in this ancestor; chromosome 5 of cow and pig are: 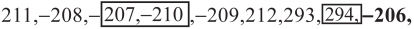 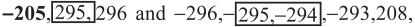 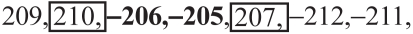 respectively, which fits the diagnostic pattern exactly.

These and other examples can, of course, be interpreted in other ways. But their existence is rather improbable without postulating transposition. This suggests that a systematic search for such patterns is warranted within a statistical model allowing a certain degree of post-transposition rearrangement.

## 9. Discussion

We have explored the small phylogeny problem under the DCJ paradigm and found that not only can it emulate MGR, and even do better in some circumstances, but by allowing circular constructs it effectively serves as a lower bound for all procedures with a constrained set of operations.

We raise the problem of the biological significance of transpositions and block interchanges and suggest that current evidence warrants a systematic study of the (existence and) prevalence of this operation.

Finally, we point out that the study of genome rearrangement is highly sensitive to the quality of the data and the degree of resolution of the procedures for demarcating conserved syntenic regions. Without a high degree of completion and correctness of genome assemblies, translocations between chromosomes may be confused with transpositions. And with increasing analytical resolution, not only do the number of conserved blocks increase, but the relative proportions of different kinds of rearrangements may shift unpredictably [6].
